# Proteomic Analysis of the Function of Sigma Factor σ^54^ in *Helicobacter pylori* Survival with Nutrition Deficiency Stress *In Vitro*

**DOI:** 10.1371/journal.pone.0072920

**Published:** 2013-08-28

**Authors:** Yundong Sun, Shuang Liu, Wen Li, Yuqun Shan, Xinpeng Li, Xingxiao Lu, Yan Li, Qing Guo, Yabin Zhou, Jihui Jia

**Affiliations:** 1 Department of Microbiology, Key Laboratory for Experimental Teratology of Chinese Ministry of Education, School of Medicine, Shandong University, Jinan, Shandong, China; 2 Clinical Laboratory, First Affiliated Hospital of Dalian Medical University, Dalian, Liaoning, China; 3 Disease Control and Prevention of Shandong Province, Jinan, China; 4 School of Control Science and Engineering, Shandong University, Jinan, China; Veterans Affairs Medical Center (111D), United States of America

## Abstract

*H. pylori* can survive under a nutrition-deficient environment. During infection and transmission, *H. pylori* is confronted with nutrient limitation and the bacterium requires rapid alteration in gene expression for survival under stress conditions. However, the mechanism underlining this regulation remains unknown. A previous study showed that σ^54^ is an important regulation factor for *H. pylori* survival in the nutrition-deficient environment. Our results show that the expression of σ^54^ (*rpoN*) is significantly induced in the stationary phase (nutrition deficiency) and the *rpoN* mutant showed a significantly lower viability than wild-type *H. pylori* in the late stationary phase. Thus, σ^54^ is involved in *H. pylori* survival during nutrient limitation. We used comparative proteomics to analyze the protein differentiation between wild-type and *rpoN* mutant during the stationary phase. With depleted nutrients, σ^54^ can slow the process of proliferation by negatively regulating genes involved in energy metabolism and biosynthesis and enhance stress-resistant ability by positively regulating genes involved in protein fate and redox reaction. Especially, NapA positively regulated by σ^54^ plays an important function in *H. pylori* survival both in the stationary phase and in water, and the latter situation would be beneficial for bacterial *in vitro* transmission. Our investigations give new light on the adaptive regulation of *H. pylori* under stress conditions.

## Introduction

*Helicobacter pylori* is a spiral-shaped, microaerophilic, Gram-negative bacterium that infects the stomachs of more than 50% of the world’s population. *H. pylori* infection is strongly associated with a spectrum of gastric diseases including chronic gastritis, peptic ulcers, and gastric cancer [1]. To establish persistent infection in the stomach, *H. pylori* must overcome constantly changing environments such as nutrient limitation, oxygen tension and low pH in the stomach. The regulatory mechanisms of *H. pylori* to allow bacterial survival under these environmental stresses are of interest.

Generally, bacterial survival under stress conditions requires timely and appropriate alterations in gene expression, and these alterations at the transcriptional level are often controlled by the association of different sigma factors with core RNA polymerase [2]. The sigma factor is an essential dissociable subunit of prokaryotic RNA polymerase that confers promoter recognition specificity on RNA polymerase in the initiation of transcription [3]. Bacteria can use alternative sigma factors, which direct the RNA polymerase holoenzyme to a specific class of promoters, to adapt to environmental changes. The association of appropriate alternative sigma factors with core RNA polymerase provides an effective mechanism for simultaneously regulating large numbers of prokaryotic genes [2]. In Gram-negative bacteria, σ^S^ is a general stress-responsive sigma factor that contributes to survival in the stationary phase and other stress conditions such as osmotic shock, heat, and low pH [4].

However, because of the small genome of *H. pylori*, relatively few transcriptional regulators for gene expression have been annotated, including just 3 sigma factors: σ^70^ (RpoD), σ^54^ (RpoN), and σ^28^ (FliA) [5]. *H. pylori* lacks σ^S^, which is typically associated with various stress responses in many Gram-negative bacteria. σ^70^, the primary (housekeeping) sigma factor, which is essential for general transcription in exponentially growing cells, has been functionally characterized in *H. pylori* [6,7]. The main function of σ^28^ in many bacterial species is to regulate the expression of genes required for flagellar synthesis and bacterial motility [8]. σ^54^ does not share a common role among all pathogens. σ^54^-dependent genes described to date control a wide variety of biological processes [4]. In *Pseudomonas aeruginosa*, σ^54^ is required for the transcription of both the flagellin and pilin genes, negatively controls quorum-sensing genes, and alters bacterial susceptibility to antibiotics [9–12]. In *Vibrio* *fischeri* , σ^54^ controls motility, biofilm formation, luminescence, and colonization [13]. In *Listeria monocytogenes*, σ^54^ is involved in resistance to high osmolarity and mesentericin Y105, an antibacterial peptide from *Leuconostoc* *mesenteroides* [14,15].

Most studies of σ^54^ in *H. pylori* have focused on its regulatory control in flagella-related genes [16]. Given the multiple regulatory controls of σ^54^ and because of the lack of σ^S^ in *H. pylori*, we were interested in whether σ^54^ of *H. pylori* could function as a stress-response sigma factor when *H. pylori* encounters environmental stresses such as nutrient limitation. We found that the expression of σ^54^ is induced when *H. pylori* enters into stationary phase. To characterize σ^54^ function, we constructed the *rpoN* null mutant. In the late stationary phase, the *rpoN* mutant *H. pylori* 26695 showed a marked decrease in viability, which agrees with a previous report in *H. pylori* HPK5 [17]. So this finding is not strain-specific and the role of σ^54^ involved in stationary phase survival might be universal in *H. pylori*. We examined the mechanism by which σ^54^ regulates *H. pylori* survival in nutrient-deficient environment by proteomics analysis through comparing the protein expression profiles of wild-type *H. pylori* 26695 and its *rpoN* null mutant in the early stationary phase.

## Materials and Methods

### Bacterial strain and growth conditions

*H. pylori* 26695 was kindly provided by Dr. Zhang Jianzhong (Chinese Disease Control and Prevention Center). The bacteria were revived from frozen stocks and grown on Skirrow agar with 5% (v/v) sheep blood under microaerobic conditions (5% O_2_, 10% CO_2_, and 85% N_2_) at 37 °C. Plate-grown bacteria were inoculated into Brucella broth (BB) containing 10% fetal bovine serum with a preliminary OD_600_ of 0.05 and cultured with shaking at 37 °C in microaerobic environment. To determine the expression of sigma factors in *H. pylori* at different growth stages, the liquid bacterial cultures were harvested at 24, 48 and 72 h for total RNA isolation.

For the viability assay, the wild type and *rpoN/npaA* mutant *H. pylori* 26695 were incubated at 37 °C microaerobically with shaking in BB containing 10% fetal bovine serum. At various times, an aliquot of each culture was serially diluted with phosphate buffered saline (PBS), and various dilutions were plated in triplicate onto agar plates at different times to determine colony formation units. To distinguish the survival ability of the wild type and *napA* mutant, an aliquot of each culture was serially diluted with PBS (preliminary OD_600_ 0.05), then put into isopyknic pure water. Each assay was replicated at least 3 times.

### Construction of the Δ*rpoN* and Δ*napA* mutant

The *rpoN* and *napA* mutant strain of *H. pylori* 26695 was constructed as described [18]. Plasmids pILL570 and pUC18K2 were kindly provided by Dr. Agnes Labigne (Institut Pasteur, Département de Microbiologie, Unité de Pathogénie Bactérienne des Muqueuses, 75724 Paris Cedex 15, France). The mutant strain was constructed in the following steps. Fragment 1 containing the 5’ region of *rpoN*(*napA*) gene flanked by *Cla*I and *Eco*RI restriction sites was amplified by PCR with the first pair of primers (*rpoN*-1 and *rpoN*-2, *napA*-1 and *napA*-2). Fragment 2 containing the 3’ region of *rpoN*(*napA*) flanked by *Bam*HI and *Pst*I restriction sites was amplified by PCR with the second pair of primers (*rpoN*-3 and *rpoN*-4, *napA*-3 and *napA*-4). *H. pylori* strain 26695 genomic DNA was used as the template and the primers are in Table 1. Following PCR amplification, fragment 1 was digested by *Cla*I and *Eco*RI and fragment 2 was digested by *Bam*HI and *Pst*I. The nonpolar kanamycin cassette was obtained from pUC18K2 by *Eco*RI and *Bam*HI digestion. Then, the resulting three fragments were ligated with the *Cla*I- and *Pst*I-digested vector pILL570 by T4 ligase (Fermentas), generating plasmid pILL570-*rpoN*(pILL570-*napA*), in which about a 1000-bp deletion of *rpoN* and *napA* were replaced by the kanamycin cassette. Finally, *H. pylori 26695* was electrotransformed with plasmid pILL570-*rpoN* (pILL570-*napA*) as described [19] and kanamycin-resistant (Kan^r^) recombinants were selected. The *rpoN*(*napA*) mutation in the Kan^r^ recombinant was verified by PCR with the primers for *rpoN*-1 and *rpoN*-4, *napA*-1 and *napA*-4.

**Table 1:** Primers Used in This Study.

*Primers*	*Sequences (5’–3’)*	*Primers*	*Sequences (5’–3’)*
*rpoN* -1	**CCATCGATGG** GAAGTCAAAACCGAATCAA	*devB* F	CGT TCG CCC ATT AGT TTG TT
*rpoN* -2	**CGGAATTC** TAAGGGGTTATCTAAAGCG	*devB* R	CAG GCA AAA GCG GAA TAA AA
*rpoN* -3	**CGGGATCC** ATACCGCCAACTGCTCAAC	*serC* F	GATGCGCTCATTCGTATCAA
*rpoN* -4	**AAAACTGCAG**GCGATCCCAAACAGAACTGC	*serC* R	CGCCATCAAATTCCCTTAAA
*napA*-1	**CCATCGAT**TTTCTCGCATGATCGGTGTA	*asd* F	GGAGCCATAGCGAGAGTTTG
*napA-2*	*CGGGATCCCGTTCAATTAGGGCATCACC*	*asd R*	*GGGCGTGGGGTAAAGATTAT*
*napA-*3	**CGGAATTC**AATCGGTGCCTTTCACATTC	*frr* F	CCTAATAACGACGGCGAAAC
*napA-*4	**AACTGCAG**AATCGGTGCCTTTCACATTC	*frr* R	TCTTGGCGGATATTCCTCAC
*16S rRNA* F	GCTCTTTACGCCCAGTGATTC	*fabE* F:	AGATTTCGTGCTTTCGCCTA
*16S rRNA* R	GCGTGGAGGATGAAGGTTTT	*fabE* R:	GCTTCTACAATGCCCACGAT
*rpoD* F	AACGCTTGGAATACAAACTGC	*ribH* F	GGGACTCCGCATTTTGATTA
*rpoD* R	GATCGGAAATCAACTTCCCTC	*ribH* R	CTTATTGCCGGCTTTACTGC
*rpoN* F	GCATTTCTTTAGCATCGCCTTAG	*pepA* F	CGGCCGATTACATGGTTACT
*rpoN* R	GAGCAGTTGGCGGTATTTGGT	*pepA* R	ATGCTCTTGCCTTCTTTGGA
*fliA* F	GGGGCGATGTTAGATTATTTG	*HP0404* F	AAGGCGAAATCCCTTGTTCT
*fliA* R	TTCCCATGCTCATTAAGGTGT	*HP0404* R	CCTGAATGCTTTGCTTAGGG
NQO3 F	AGA ATG CAC CCA CAA ACT CC	*sodB* F	AATCTCATCAAAGGCACG
NQO3 R	TCG CAT TCT TTA GCG CTC TTT	*sodB* R	GCTTAGGCAATCCCAATA
*acnB* F	TTAGGGATAATGCGGTCGTC	*napA* F	TGAAGAGTTTGCGGACAT
*acnB* R	GGGATTCGCCCTAGTAAAGC	*napA* R	AGAGTGGAAGCTCGTTTT
*trx*2F	ACGCAGAGAAAATCGCTCAT	*napA*ExF	TACTCAGAATTCAAAACATTTGAAATTCTAAAACAT
t*rx*2R	CTACAAGCCTTTCCCCCACT	*napA*ExR	TACTCACTCGAGGAATTTAAAGAGCTCTC

F: forward primer; R: reversed primer

Bold letters indicates nucleotides that were added at 5’ end to create a restriction site.

Restriction sites for ClaI (*rpoN*-1/*napA-1*), EcoRI (*rpoN*-2/*napA-3*), BamHI (*rpoN*-3/*napA-2*)

and PstI (*rpoN*-4/*napA-4*) are italics

### Confocal microscopy

To determine bacterial shape and survival ability, bacterial cells were stained with membrane-permeant and -impermeant fluorescent dye with LIVE/DEAD BacLight Bacterial Viability Kits (Molecular Probes, Invitrogen, USA) followed by confocal microscopy. *H. pylori* cells were collected, washed, and resuspended in BB liquid medium, then inoculated to the desired optical density at OD_600_ into BB liquid medium buffered with 10 mM sodium phosphate (pH 6.3) and supplemented with 10% newborn calf serum or pure water (preliminary OD_600_ 0.05) and grown under microaerobic conditions. Aliquots taken at different time points were stained with SYTO 9 and propidium iodide (PI) for 15 min, and washed twice with PBS. Cells were then spread on slide glasses, covered with mounting medium and cover slips, and visualized by confocal microscopy (Leica TCS SP5; Leica Microsystems GmbH, Wetzlar, Germany). SYTO 9 is a green fluorescent membrane-permeant dye that labels all bacteria by staining nucleic acid, whereas PI is a red fluorescent membrane-impermeant dye that labels only bacteria with damaged membranes.

### Quantitative real-time RT-PCR

Total cellular RNA was isolated from harvested bacteria by use of TRIzol reagent (Invitrogen). Then cDNA was obtained by use of the Revert Aid First Strand cDNA Synthesis Kit (Fermentas). The primers for PCR are in Table 1. An amount of 10 μl SYBR Premic *Ex* Taq (Takara) and 0.4 μl ROX Reference Dye (Takara) were contained in each 20 μl PCR reaction mixture. Then real-time RT-PCR involved use of the ABI Prism 7000 Sequence Detection System (Applied Biosystems) with the following protocol: one cycle at 95 °C for 10 s, 40 cycles at 95 °C for 5 s and 60 °C for 31 s. A melting curve analysis for each PCR reaction was performed after amplification to ensure the purity of the amplification product. The data were normalized by 16S rRNA expression in each sample and 3 biological replicates were performed. In this study, the 2^-ΔΔCt^ method was used as the quantitative method in real-time PCR.

### Preparation of bacterial cell lysates and 2-D gel electrophoresis (2-DE)

The wild-type and *rpoN* mutant were inoculated into BB containing 10% fetal bovine serum with a preliminary OD_600_ of 0.05 and cultured for 48 h. Then bacterial cells were harvested by centrifugation at 5,000×*g* for 10 min at 4 °C, washed 3 times with ice-cold PBS (pH 7.4) and solubilized in lysis buffer containing 8 M urea, 2 M thiourea, 4% CHAPS, 1% DTT, 1% pharmalyte (pH range 3~10), 1% protease inhibitor, and 1% nuclease mix (Amersham biosciences). After sonication (Vibra cell sonicator; 70% power, 2-s on/1-s off pulses, 5 min total), the solution was centrifuged at 20,000×g for 60 min at 4 °C and the insoluble material was discarded. The protein concentration was then measured by the Bradford method.

For isoelectric focusing, approximately 300 μg protein was adjusted to a total volume of 340 ml with rehydration buffer (containing 8 M urea, 4% CHAPS, 20 mM DTT, 0.5% Pharmalyte and a trace of bromophenol blue) and loaded onto 18 cm immobilized pH gradient (IPG) strip (pH 3–10 NL). The strips were rehydrated on an IPGphor instrument (Amersham Biosciences) for 12 h at 60 V. Then the voltage was set for 2 h at 100 V and 1 h at 500, 1000 and 5000 V and kept at 8000V until a total of 80 kVh. Subsequently, IPG strips were equilibrated for 15 min each in buffer (50 mM, Tris-HCl; pH 8.8, 6 M urea, 30% glycerol, 2% SDS, a trace of bromophenol blue) with 0.5% (w/v) DTT and 2% (w/v) iodoacetamide. For 2D separation, the strips were transferred onto 12% uniform SDS–PAGE gel, followed by electrophoresis conducted at 15 mA each gel for 30 min and then 30 mA each gel until the bromophenol blue dye reached the bottom of the gel by use of the PROTEAN II xi 2-D cell (Bio-Rad). The gels were silver-stained and scanned by use of ImageScanner II (Amersham biosciences) at 256 grayscale and 300 dpi. 2-DE was repeated 3 times with independently grown cultures. Image analysis involved use of ImageMaster 2D Elite 5.0 (Amersham Biosciences). The proteome profile of wild-type *H. pylori* 26695 was used as a reference for spot analysis, and a 2-fold change (*P* < 0.05) in spot volume was defined as the significant change.

### In-gel digestion and MALDI-TOF/TOF MS

Protein spots showing differential expression on 2-DE gels were excised manually, then digested with trypsin and desalted by passing through a C18 ZipTip (Millipore, USA). The resulting peptides were mixed with *a*-cyano-4-hydroxycinnamic acid and spotted onto MALDI target plates. Peptide mass fingerprints were obtained by use of a MALDI-TOF/TOF-tandom mass spectrometer (Applied Biosystems). The MS and MS/MS spectra were analyzed with a 50 ppm mass tolerance by use of GPS Explorer V.2.0.1 and Mascot V 1.9 based on NCBI SWISSPROT and local *H. pylori* databases (April 2006 updated).

### Recombinant expression and purification of NapA and antiserum preparation

From the *napA* complete cDNA sequence, we designed a pair of primers, *napA*ExF and *napA*ExR, to amplify the sequence encoding the mature peptide. The obtained fragment was digested by *EcoR*Ⅰand *Xho*I and inserted into the pTriEx-4 expression vector (Novagen, USA) that contains a 6-Histidine-tag (His-tag) that is supposedly fused with recombinant NapA. The recombinant plasmid was transformed into competent *Escherichia coli* BL21(DE3) cells for NapA protein overexpression. After induced expression with isopropyl-β-D-thio-galactoside at 28 °C, the bacterial pellets were collected by centrifugation and were re-suspended in PBS containing 1% Triton X-100 for probe sonication lysis. The recombinant NapA protein was purified by use of a His-bind column (Novagen, USA) following the instructions of the manufacturer. The antiserum was prepared with the purified recombinant NapA protein as described [20].

### Western blot analysis of NapA

The wild-type and *rpoN* mutant *H. pylori* 26695 were inoculated into BB containing 10% fetal bovine serum with a preliminary OD_600_ of 0.05 and cultured for 24, 48 and 72 h. Then bacterial cell lysates were prepared as described previously. The Bradford method was used to determine protein concentration, and approximately 25–30 μg protein of each sample was loaded and separated by 12.5% SDS–PAGE. The proteins in the gel were then transferred onto a nitrocellulose membrane, which was blocked in 3% non-fat milk in TBS (10 mM Tris–HCl and 150 mM NaCl; pH 7.5), then incubated with the polyclonal antibody against NapA, washed 3 times with TBS, then incubated with secondary antibody (peroxidase-conjugated goat anti-rabbit IgG; 1/10,000 TBS dilution). Finally, the NapA protein was visualized in a colorimetric reaction.

### Statistical Analysis

Data are presented as mean ± SEM. Statistical significance was determined by unpaired Student’s *t* test and one-way ANOVA. *p* < 0.05 was considered statistically significant. Results were analysed by use of Graphpad Prism (Graphpad Software Inc, La Jolla, CA, USA). Self-organizing map (SOM) analysis by the MATLAB 6.5 environment was used for SOM training. Illustration of SOM outputs by bar-graph display or component-plane presentations (CPP) was conducted in the MATLAB 6.5 environment (www.mathworks.com).

## Results

### σ^54^ is involved in *H. pylori* 26695 survival during the stationary phase

**Figure 1:**
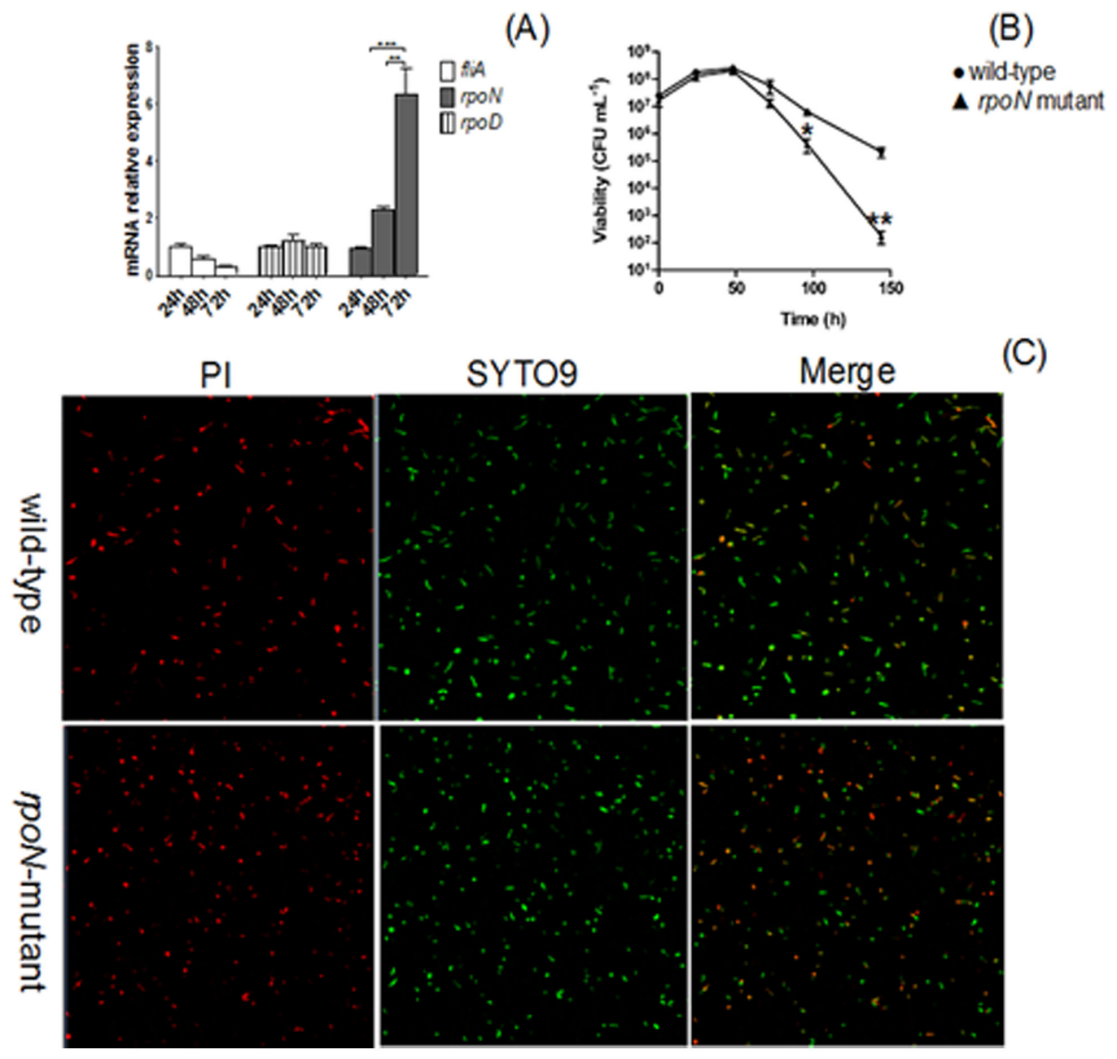
*RpoN* is involved in *H. pylori* 26695 survival during the stationary phase. (A) RT-PCR analysis of the mRNA levels of sigma factors in *H. pylori* 26695 at different growth stages. Data are mean ± SEM from 3 independent experiments. (B) The survival of *ΔrpoN* strain was deficient during the stationary phase. Bacteria were cultured in Brucella broth containing 10% fetal bovine serum, and numbers of colony formation units (CFU) per milliliter were determined at the times indicated. A representative assay of at least 3 trials is shown. Data are mean ± SEM from 3 independent experiments. (C) Lack of nutrition induces coccoid transformation and death of *H. pylori* cells. Wild-type and *ΔrpoN* mutant strain were cultured in liquid medium for 144 h. Cells stained with membrane-permeant SYTO 9 (green) and membrane-impermeant PI (red) were visualized by confocal microscopy. Data are representative of 3 independent experiments.

The mRNA level of *rpoN* (σ^54^) was significantly induced (*P*<0.05) in *H. pylori* 26695 in the stationary phase of culture (48 and 72 h) as compared with the exponential phase (24 h) (Figure 1A). In contrast, the expression of *fliA* and *rpoD* did not significantly change during the transition from the exponential to stationary phase (Figure 1A).

To clarify whether *rpoN* is required for *H. pylori* survival, we constructed the *rpoN* null mutant, and then examined the growth curves of wild-type and *rpoN* mutant over 6 days. The *rpoN* mutant exhibited a marked decrease in viability as compared with wild-type *H. pylori* in the late stationary phase (Figure 1B). Furthermore, we examined *H. pylori* cell membrane integrity and morphologic features of the wild-type and *rpoN* mutant under nutrient-deficient conditions with membrane-permeant (SYTO 9) and membrane-impermeant PI fluorescent dyes, staining living and dead cells respectively. More *rpoN*-null cells lost cytoplasmic membrane integrity than did wild-type cells and transformed from its normal helical bacillary morphologic features to coccoid features after 144 h of nutrition deprivation (Figure 1C).

The above results indicate that σ^54^ indeed takes part in the nutrient-deficient response of *H. pylori*, so we investigated its regulation mechanism.

### Comparison of differential expression of protein between wild-type and *rpoN* mutant *H. pylori*

**Figure 2:**
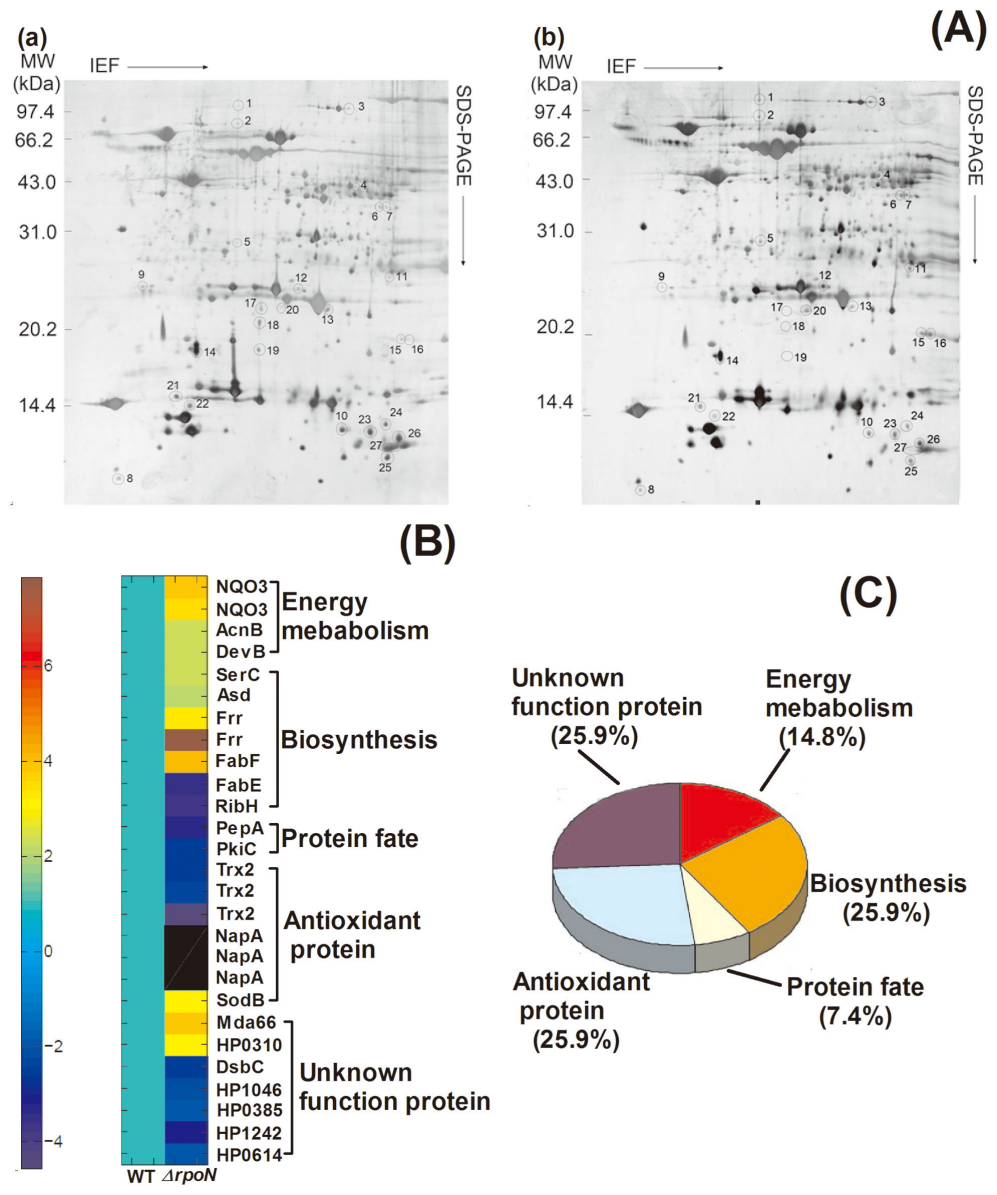
Proteomics analysis of proteins with differential expression between wild-type and *ΔrpoN H. pylori* 26695. (A) Representative 2-DE maps of wild type (a) and*ΔrpoN* mutant (b) in the early stationary phase. IEF, isoelectric focusing. Protein spots showing significant changes between the 2 strains are indicated by circles and numbers. (B) Hierarchical clustering analysis of the 27 differentially expressed proteins; On the scale bar, blue and red represent the degree of down- and up-regulation of protein abundance, respectively. (C) Gene ontology classification of the 27 proteins with differential expression.

**Figure 3:**
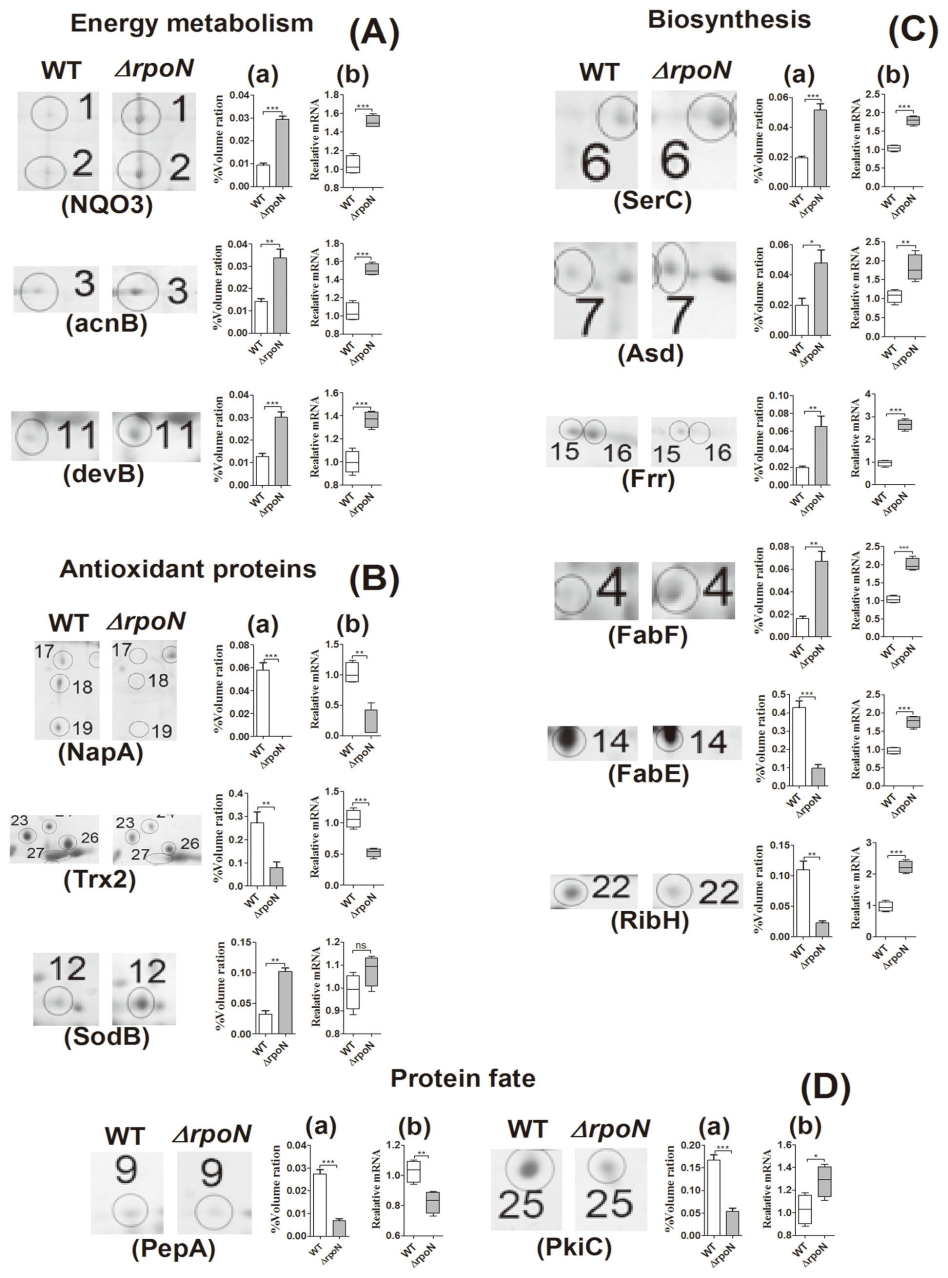
mRNA expression confirms the protein expression of most genes from 2-DE maps. mRNA and protein levels of the wild-type (WT) and Δ*rpoN* involved in energy metabolism (A), antioxidation (B), biosynthesis (C) and protein fate (D). (a) Quantification of total protein spot volume ratio from 2-DE analysis; (b) RT-PCR analysis of mRNA levels. Box-and-whisker plots represent the median value with 50% of all data falling within the box. The “whiskers” extend to the 5th and 95th percentiles. Signals were normalized to 16S rRNA levels. Data are mean ± SEM of replicated experiments. *P <0.05, **P <0.01, ***P <0.001.

We used proteomic analysis to compare the protein expression profiles of *H. pylori* 26695 and its *rpoN* null mutant in the early stationary phase to determine the mechanism by which σ^54^ regulates *H. pylori* survival. Compared with the proteome of wild-type *H. pylori*, 27 protein spots were differentially expressed in the *rpoN* mutant (Figure 2A; Table 2). The functions of these proteins were involved in energy metabolism, biosynthesis, redox reaction, protein fate, and so on (Table 2; Figure 2B and C). The differential expression of these proteins was further confirmed at the mRNA level by quantitative real-time PCR (Figure 3)*.*

**Table 2:** Summary of protein spots showing significant changes in the 2-DE maps of wild-type *H. pylori* 26695 and its *rpoN* mutant.

***Annotation***	***Spotno.*^*a*^**	***Genesymbol***	***TIGRORFno.*^*b*^**	**Fold-change^c^**
**Energy metabolism**				
Oxidative phosphorylation/Nitrogen metabolism	1	*NQO3*	HP1266	+3.83
	2	*NQO3*	HP1266	+3.83
TCA cycle	3	*acnB*	HP0779	+2.35
Pentose phosphate metabolism	11	*devB*	HP1102	+2.30
**Biosynthesis**				
Amino acid biosynthesis	6	*serC*	HP0736	+2.41
	7	*asd*	HP1189	+2.24
Riboflavin biosynthesis	22	*ribH*	HP0002	-3.68
Protein synthesis	15	*frr*	HP1256	+3.23
	16	*frr*	HP1256	+7.87
**Protein fate**				
Glutathione metabolism	9	*pepA*	HP0570	-3.34
Protein stability	25	*SP:P16436*	HP0404	-2.56
**Antioxidant proteins**				
Thioredoxin, glutaredoxin, and glutathione	23	trx2	HP1458	-2.56
	26	trx2	HP1458	-2.30
	27	trx2	HP1458	-4.55
Detoxification	17	*napA*	HP0243	Absent
	18	*napA*	HP0243	Absent
	19	*napA*	HP0243	Absent
	12	*sodB*	HP0389	+3.16
	20	*mda66*	HP0630	+3.84
**Unknown function protein**	5	*chp* ^*d*^	HP0310	+3.06
	13	*dsbC*	HP0377	-2.49
	21	*chp* ^*d*^	HP1046	-2.13
	8	*chp* ^*d*^	HP0385	-2.00
	10	*chp* ^*d*^	HP1242	-3.09
	24	*chp* ^*d*^	HP0614	-2.00

a Spot numbers refer to the proteins labeled in Figure 2.

b TIGR ORF no. follows the nomenclature of *H. pylori* strain 26695.

c Fold-change for each protein derived from isogenic *rpoN* mutant compared with the protein derived from wild-type *H. pylori* 26695.

d *chp*: conserved hypothetical protein

### NapA recombinant expression, purification, and polyclonal antibody preparation

**Figure 4:**
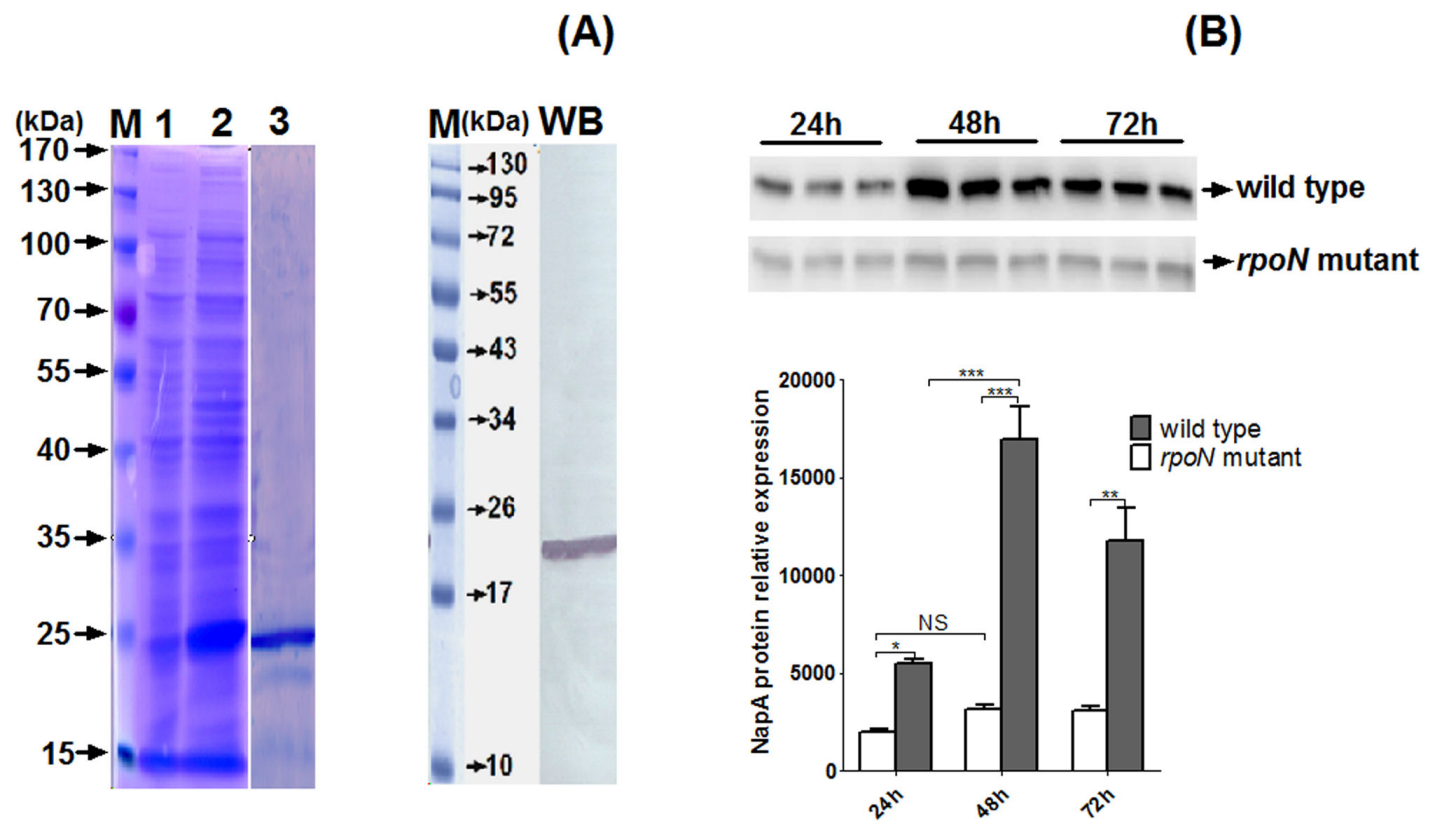
The protein expression of NapA was decreased inΔ*rpoN* strain from the exponential to stationary phase. (A) Expression and purification of recombinant NapA. Lane 1: Total protein of *E. coli* with pTriEx-4/NapA, without induction; Lane 2: Total protein of *E. coli* with pTriEx-4/NapA, with isopropyl-β-D-thio-galactoside induction; Lane 3: purified recombinant NapA; WB: Western blot analysis of the polyclonal antibody of NapA. The band shows that the recombinant NapA was recognized by polyclonal antibody. (B) NapA protein expression in wild-type and Δ*rpoN* strain from the exponential to stationary phase. The graph represents the relative NapA protein expression from western blot analysis.

We performed recombinant expression and purification of NapA. The deduced molecular weight of recombinant NapA was approximately 24 kDa, including a 7-kDa 6-Histidine-tag expressed by the plasmid pTriEX-4, which was generally consistent with the SDS-PAGE results (Figure 4A). Western blot analysis revealed only 1 band, whose size was in agreement with the predicted molecular weight of the recombinant NapA (Figure 4A). Therefore, the prepared antiserum could uniquely recognize recombinant NapA.

### Expression of NapA was positively regulated by σ^54^

The expression of NapA was absent in the *rpoN* mutant during the stationary phase (Table 2 and Figure 2), so NapA was positively regulated by σ^54^. We confirmed this result with western blot analysis (Figure 4B). The protein level of NapA was gradually increased in the wild-type strain and quickly reduced in *rpoN* mutant from the exponential to stationary phase (Figure 4B).

### *napA* null mutant survival was decreased under nutrient deficiency

**Figure 5:**
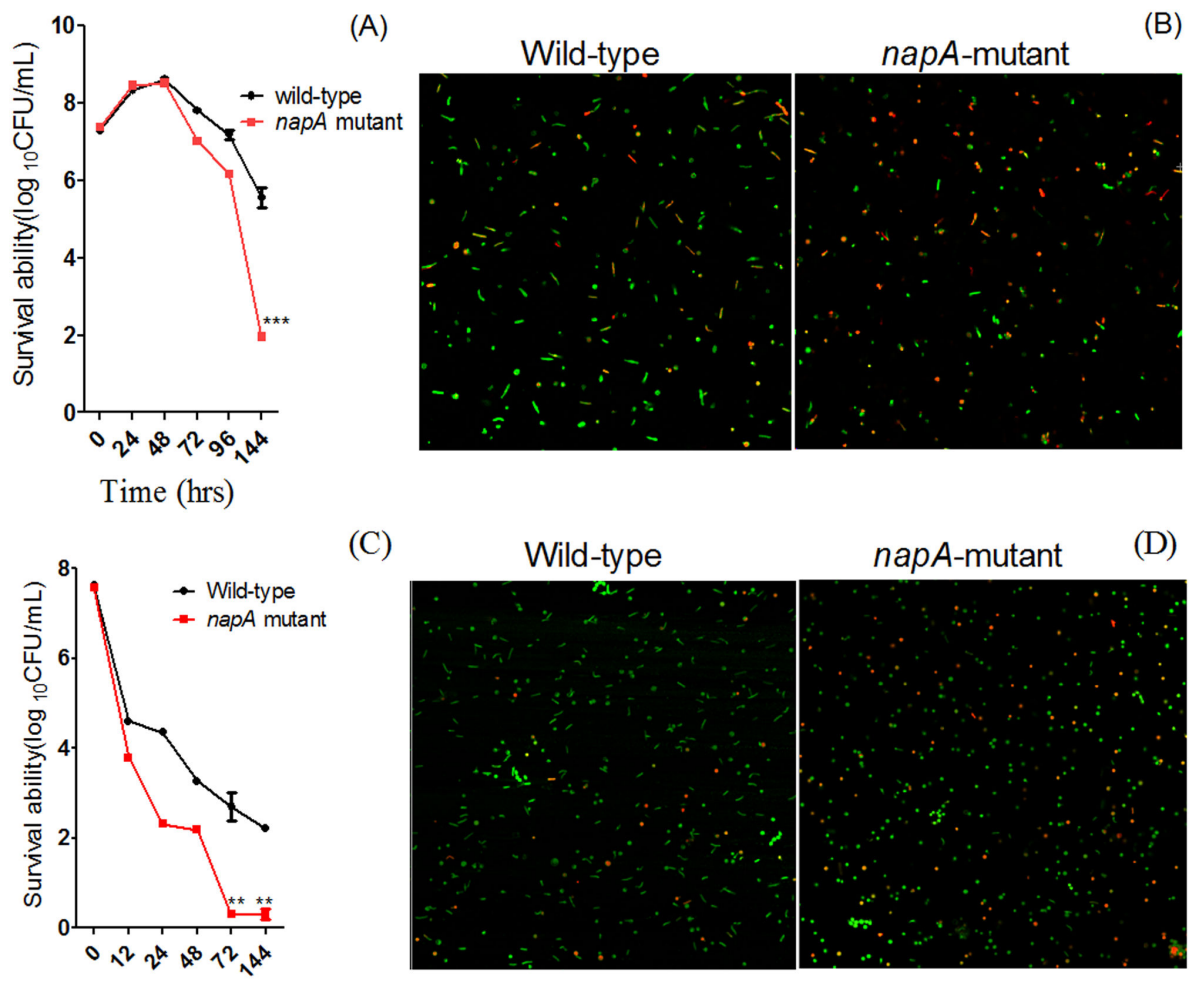
NapA is involved in *H. pylori* 26695 survival during the stationary phase and in water. (A) The survival of *ΔnapA* strain was deficient during the stationary phase. Bacteria were cultured in Brucella broth containing 10% fetal bovine serum, and numbers of CFUs per milliliter were determined at the times indicated. (C) The survival of the *ΔnapA* strain was deficient in water. Bacteria during the exponential phase were placed in pure water and cultured in an enclosed environment, and numbers of CFUs per milliliter were determined at the times indicated. A representative assay of at least 3 trials is shown. Data are mean ± SEM from 3 independent experiments. (B) Lack of nutrition induces coccoid transformation and death of *H. pylori* cells. Wild-type and *ΔrpoN* mutant were cultured in liquid medium for 144 h. (D) Inoculation into water induces coccoid transformation and death of *H. pylori* cells. Wild-type and *ΔnapA* mutant survived in water. Cells stained with membrane-permeant SYTO 9 (green) and membrane-impermeant PI (red) were visualized by confocal microscopy. Data are representative of 3 independent experiments.

To determine whether NapA protein takes part in *H. pylori* survival in a nutrient-deficient environment, we constructed the *napA* null mutant and examined the growth curves of the wild-type and *napA* mutant *H. pylori* during the stationary phase and in water. The *napA* mutant showed a marked decrease in viability as compared with wild-type *H. pylori* (Figure 5A/C) both in the stationary phase and in water. Additionally, most *napA* mutants transformed from the normal helical bacillary features to coccoid features (Figure 5B/D).

## Discussion

*H. pylori* inevitably responds to nutrient-deficient stress during *in vitro* transmission, but studies of this observation are few, especially the molecular mechanism. Nutrient limitation also occurs when the bacteria enters the stationary phase, because their rapid growth and efficient metabolism in the exponential phase ultimately results in depletion of growth-supporting substrates and nutritional shift-down. To survive in this starved condition, *H. pylori* requires rapid alterations in gene expression to adapt to an unfavorable environment. We found that the expression of *rpoN* was significantly increased when *H. pylori* entered the stationary phase (Figure 1A), and the survival of the *rpoN* mutant strain was weakened (Figure 1B,C). These results confirm the previous findings [17] and imply that σ^54^ has an important function in *H. pylori* survival with limited nutrition. However, the underlying mechanism is still not clear. Thus, we compared the protein expression pattern of the wild-type and *rpoN* mutant strain during the stationary phase. Proteomic analysis identified 11 proteins downregulated and 10 proteins upregulated by σ^54^ in stationary phase. The differential expression of these proteins was further confirmed at the mRNA level (Figure 3). Surprisingly, the expression of 3 proteins (FabE, RibH, and PkiC) conflicted with their mRNA levels. The protein levels of these 3 genes were decreased while their mRNA levels were increased in the *rpoN* mutant as compared with wild-type *H. pylori*. Certain regulators might be activated directly or indirectly in the *rpoN* mutant and post-transcriptionally regulate these genes. mRNA processing and decay in prokaryotes can be controlled by post-transcriptional regulation [21], and post-transcriptional regulatory mechanisms may be important in facilitating the response of *H. pylori* to diverse environmental stimuli [22–24].

According to proteomic analysis, some proteins were significantly increased in the *rpoN* mutant relative to wild-type *H. pylori* (Figure 2, Table 2). The expression of 3 enzymes (aconitase B, NADH-ubiquinone oxidoreductase NQO3 subunit, glucose-6-phosphate 1-dehydrogenase) involved in energy metabolism was increased in the *rpoN* mutant. Additionally, the expression of 4 proteins involved in biosynthesis was increased, including that of 2 enzymes (phosphoserine aminotransferase, aspartate-semialdehyde dehydrogenase), responsible for amino acid biosynthesis; 1 protein (ribosome releasing factor), participating in protein synthesis; and beta ketoacyl-acyl carrier protein synthase II, related to fatty acid biosynthesis. During the stationary phase, when growth ceases, bacteria have developed a strategy to balance the need to proliferate with the need to protect themselves against stress [25]. Our data indicate that σ^54^ negatively regulates proteins related to energy metabolism and biosynthesis with deficient nutrients, and this control would permit the bacteria to adjust themselves to nutritional stress for increased survival under this condition. A previous study showed that disruption of *acnA* encoding aconitase enhanced the survival of stationary-phase *Staphylococcus aureus* cells about 100-fold [26].

Protein degradation has an important function for bacteria survival in nutrition-deficient environments [27]. For example, ClpP-containing proteases are critical in stationary-phase adaptation of *E. coli* [28]. It can be seen from the Figure 2 that the proteins taking part in protein fate were decreased in level in the *rpoN* mutant during the stationary phase. Aminopeptidase a/I and protein kinase C inhibitor (SP: P16436), 2 proteins involved in protein fate, are responsible for degradation of proteins, peptides and glycopeptides, which would facilitate amino acid recycling under nutrient-deficient situations to increase the survival of bacteria.

The redox protein thioredoxin and the associated enzyme TrxR constitute a thiol-dependent reduction-oxidation system that can catalyse the reduction of specific proteins (e.g., a number of reactive oxygen species detoxification enzymes) by NADPH [29]. In our study, the expression of thioredoxin was downregulated in the *rpoN* mutant. Mutation of thioredoxin in *H. pylori* resulted in increased sensitivity to several forms of oxidative and nitrosative stress [30]. Previous study revealed that oxidative damage may be the Achilles’ heel of stationary-phase bacterial cells and cells subjected to nutrient starvation would have increased demand for oxidation management [31]. NapA plays an important role in protecting *H. pylori* against oxidative stress, and the survival of the *napA*-null *H. pylori* mutant was decreased in the presence of oxidative stress [32]. We found that the expression of NapA was decreased in the *rpoN* mutant but increased in the wild-type strain in the stationary phase (Figure 2 and Figure 4B). Furthermore, the survival of the *napA* mutant was weakened as compared with the wild-type strain during the stationary phase (Figure 5A). Our study indicated that NapA was positively regulated by σ^54^, which would benefit *H. pylori* to defend against oxidative damage in the stationary phase and enhance survival under nutritional starvation. Moreover, the survival of the *napA* mutant was weakened in water as compared with the wild-type strain (Figure 5C), and most of the *napA* mutants become coccoid (Figure 5D). The *H. pylori* non-culturable coccoid form was previously found in tap water kept at 4°C for 7 days [33], so water is one of the risk factors of *H. pylori* transmission and infection *in vitro* [34,35]. Positive regulation of NapA by σ^54^ would be helpful for prolonged survival of *H. pylori* in water, which may play an important role in *H. pylori* transmission and infection.

However, a previous report noted that NapA production is under the control of ferric uptake regulator (Fur), which behaves as a specific repressor or as a more general regulator under oxidative stress [32], whereas our study indicated that NapA was positively regulated by σ^54^ during nutritional deficiency. Thus, further experimental confirmation is required to evaluate the relative contributions of Fur and σ^54^ on regulating NapA production in *H. pylori* under certain unfavorable conditions.

Unexpectedly, we found that the expression of 2 antioxidant proteins, superoxide dismutase and modulator of drug activity, was elevated in the *rpoN* mutant. This increase might be a compensatory response by the *rpoN* mutant in the stationary phase to combat oxidative stress, because the expression of other important antioxidant proteins (thioredoxin and NapA) was decreased in the *rpoN* mutant. Consistent with our presumption, modulator of drug activity was found as a novel potential antioxidant by its upregulation compensating for the loss of other major antioxidant components [36].

Additionally, the expression of 6 proteins with unknown function was decreased in the *rpoN* mutant with nutrition deficiency. Understanding the roles of these proteins could provide further insights into the σ^54^ function in *H. pylori*.

## Conclusions

The present work reveals that σ^54^ is involved in *H. pylori* survival in the stationary phase. We describe a simple model of the regulation of σ^54^ on *H. pylori* survival of stationary phase (nutritional stress). Proteomic analysis revealed that σ^54^ can decrease the need for proliferation by negatively regulating the genes involved in energy metabolism and biosynthesis and enhance stress-resistant ability by positively regulating the genes involved in protein fate and redox reaction, thus resulting in prolonged survival in a nutrient-starved environment. Our investigation provides new insight into the adaptive regulation of *H. pylori* under stress conditions.
